# In Vitro Validation of an Artefact Suppression Algorithm in X-Ray Phase-Contrast Computed Tomography

**DOI:** 10.1371/journal.pone.0135654

**Published:** 2015-08-21

**Authors:** Naoki Sunaguchi, Tetsuya Yuasa, Shin-ichi Hirano, Rajiv Gupta, Masami Ando

**Affiliations:** 1 Graduate School of Engineering, Gunma University, Kiryu, Gunma, Japan; 2 Graduate School of Engineering and Science, Yamagata University, Yonezawa, Yamagata, Japan; 3 Mercian Cleantec Corporation, Fujisawa, Kanagawa, Japan; 4 MiZ Corporation, Kamakura, Kanagawa, Japan; 5 Department of Radiology, Massachusetts General Hospital and Harvard Medical School, Boston, Massachusetts, United States of America; 6 Research Institute for Science and Technology, Tokyo University of Science, Noda, Chiba, Japan; University of Nebraska Medical Center, UNITED STATES

## Abstract

X-ray phase-contrast tomography can significantly increase the contrast-resolution of conventional attenuation-contrast imaging, especially for soft-tissue structures that have very similar attenuation. Just as in attenuation-based tomography, phase contrast tomography requires a linear dependence of aggregate beam direction on the incremental direction alteration caused by individual voxels along the path of the X-ray beam. Dense objects such as calcifications in biological specimens violate this condition. There are extensive beam deflection artefacts in the vicinity of such structures because they result in large distortion of wave front due to the large difference of refractive index; for such large changes in beam direction, the transmittance of the silicon analyzer crystal saturates and is no longer linearly dependent on the angle of refraction. This paper describes a method by which these effects can be overcome and excellent soft-tissue contrast of phase tomography can be preserved in the vicinity of such artefact-producing structures.

## Introduction

In conventional X-ray imaging, image contrast arises from attenuation of X-rays due to photoelectric absorption and Compton scattering. All clinical X-ray imaging systems currently deployed in medical practice use such attenuation as their primary source of image contrast. Because the attenuation contrast is sensitive to difference in atomic number, tissues that are markedly different from their background are well seen in conventional X-ray imaging. For example, bone pathology such as bone tumours and fractures are well depicted by X-ray radiography and computed tomography (CT). On the other hand, soft tissue abnormalities may be hard to detect using these modalities because attenuation of different types of soft tissues is very similar [1]. For example, various components of atherosclerotic plaque such as fibrous cap and atheroma are essentially indistinguishable at catheter angiography and CT.

X-ray phase-contrast imaging (XPCI) explores an alternative mechanism of interaction between the X-ray wave and tissue, namely phase alteration or bending of X-rays due to electron clouds of various materials [2]. Even though different types of soft tissues have very similar attenuation, they have widely varying refractive index that is responsible for markedly different phase alterations imparted by different types of soft tissues. This can be picked up by a variety of phase sensitive methods such as X-ray interferometry, in-line holography, angle analyzer in crystal optics and grating optics. As a result, XPCI can provide high image contrast for soft tissue structures [2]. A variety of novel XPCI systems have been demonstrated both using synchrotrons [3–7] as well as laboratory-scale X-ray sources [8–12].

**Fig 1:**
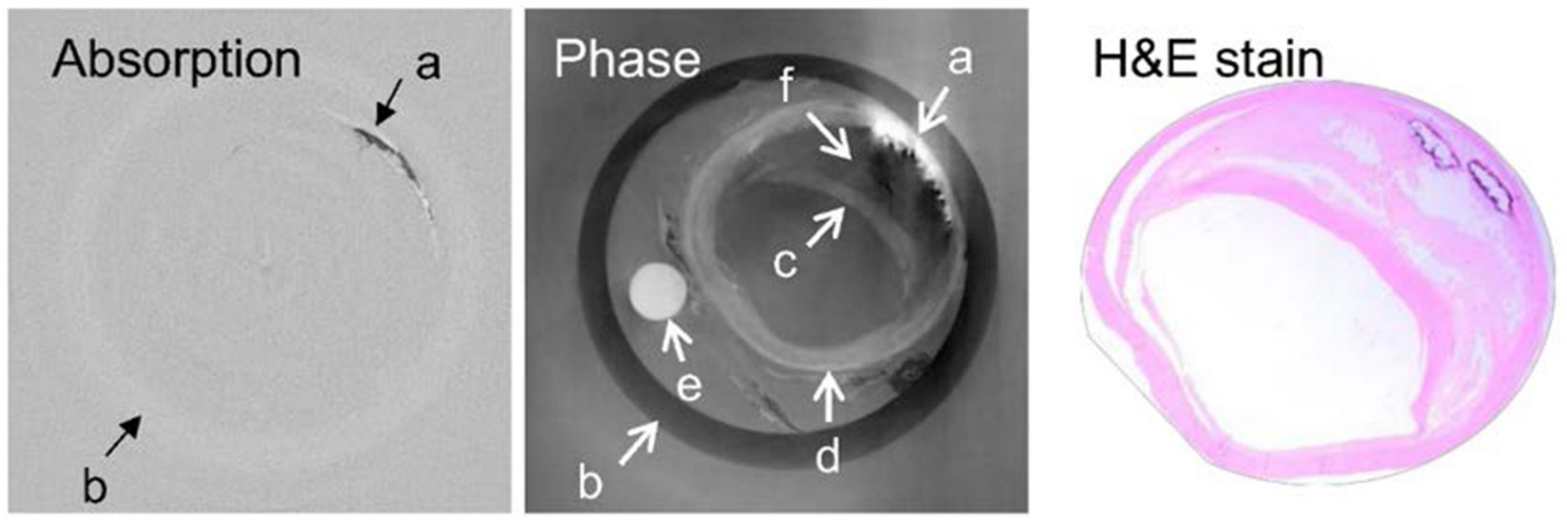
Improved soft-tissue contrast afforded by X-ray phase images: An iliac artery with extensive atherosclerotic disease demonstrating different plaque components in the phase but not attenuation image a: calcification; b: plastic container; c: fibrous cap; d: tri-laminar arterial wall; e: a plastic rod as fiducial marker; f: soft plaque or atheroma.

It is widely acknowledged that XPCI improves soft-tissue contrast of traditional attenuation-based X-ray imaging [2]. For example, we have shown that XPCI can characterize atherosclerotic plaque and discriminate benign from cancerous tissue [13] (Fig 1). All systems currently in use are only suitable for small ex-vivo specimens and small animals. Considerable efforts are being made to scale up the size of these phase-contrast imaging systems for human imaging [14, 15].

A key technical limitation of analyzer-based phase imaging systems is that they cannot accurately reconstruct a tomographic slice when the X-ray propagation suffers a large change in the direction. The reason for this limitation is as follows: Phase-contrast X-ray tomography, just as in conventional attenuation-based tomographic reconstructions, requires that the intensity in the projection images represent a Radon transform of the differential refractive index map of the object being imaged. As explained below, the intensity in the projection images varies with the beam deflection only for a limited range. Within this range, the propagation direction falls within the measurable region of the rocking curve of analyzer in crystal optics. However, for large direction change at tissue boundary, this condition breaks down because the direction deviates from the measurable region of the analyzer, resulting in a degenerate condition.

Soft-tissue structures are weakly refracting because the real part of the their complex refractive index, the component that is responsible for phase alteration of the wave front, is very close to 1.0 for X-rays. Therefore, they cause only a small amount of bending in the x-ray wave front and the relationship between the intensity and angle of refraction is one-to-one. As a result, an accurate phase map can be obtained for soft tissues by applying inverse Radon transform to the acquired projection images. On the other hand, dense structures such as bones and calcifications result in a large change in direction and violate the one-to-one condition: The amount of phase change can no longer be properly reproduced from the intensity in the acquired phase projection using the rocking curve. As a result, when standard methods for obtaining the inverse Radon transform are applied, the resulting phase image has extensive artefacts in the vicinity of dense structures. In their genesis, these artefacts are similar to the metal and beam-hardening artefacts seen in traditional attenuation-based X-ray computed tomography even though they tend to be more severe and have a different physical basis.

Fig 1 provides an example of this artefact resulting from the large alteration of the wave propagation direction. As can be seen, all information in and around the dense calcification in the top right quadrant of this artery specimen is completely destroyed. The absorption or attenuation image, on the other hand, depicts calcification well and this artefact is present only in the phase image. Since the attenuation image provides a map of the areas where the voxels responsible for phase aberrations reside, one can potentially suppress them using an iterative tomographic reconstruction algorithm. A preliminary reconstruction algorithm for overcoming this artefact in the vicinity of dense objects based on this idea was presented in [16]. The algorithm presented in [16] iteratively processes the phase and the attenuation image in the Radon space in order to reconstruct the missing information. A drawback of this algorithm is that noise accumulates with each iterative step. As a result, severe microscopic artefacts, which worsen with each iterative step, appear in the resultant reconstructed image. In order to reduce these artefacts, we have incorporated a de-noising step based on the total variation in the algorithm presented in this paper. We have also validated the efficacy of this algorithm on three in-vitro biological specimens that contain artefact generating dense components.

The remainder of this paper is organized as follows. We first present the details of the experimental setup used for phase contrast imaging and the iterative algorithm with denoising procedures for tomographic phase image reconstruction. The efficacy of this algorithm was validated on three biological specimens. The steps taken for sample preparation and for acquiring the images are detailed in the *Methods and Materials* section. The *Results* section shows the phase images with and without the new artefact reduction algorithm. Discussion and concluding remarks are presented in the last section.

## Materials and Methods

### X-ray Dark-field Imaging (XDFI) System

**Fig 2:**
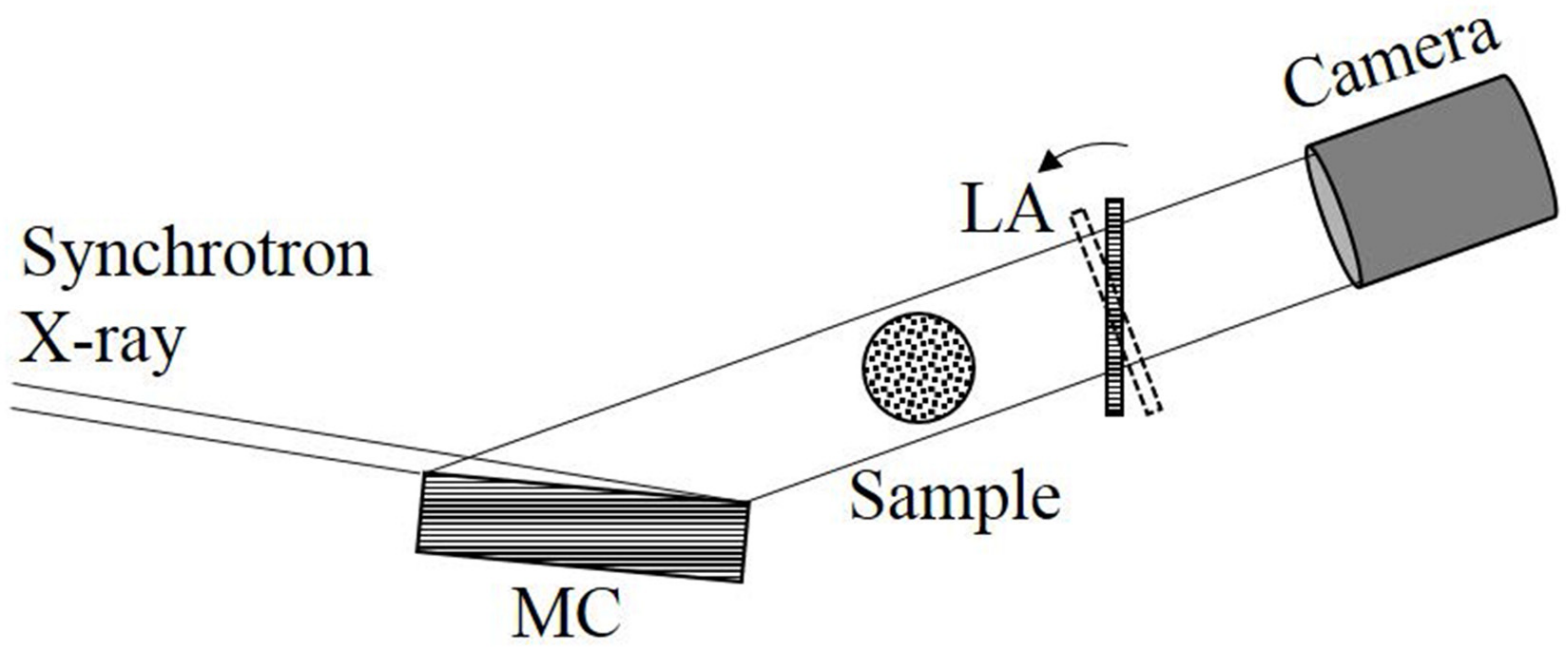
Schematic representation of the XDFI system.

A schematic of XDFI system is shown as Fig 2. The system comprises a synchrotron X-ray source from 2.5 GeV storage ring at the KEK Photon Factory (Tsukuba, Japan), an asymmetrically cut Bragg-case monochromator-collimator (MC), a sample rotational stage, a Laue-case angle analyser (LA), and an X-ray CCD camera. As X-ray beam from the synchrotron that has already been monochromated by a double-crystal monochromator is incident on MC with an angle (*θ*_B_ − *α*), where *θ*_B_ is the Bragg angle and *α* is the asymmetric angle between the crystal surface and the diffracting plane. In this process, the asymmetrically cut MC expands the X-ray beam size by a factor of 1/*b* = sin(*θ*_B_ + *α*) / sin(*θ*_B_ − *α*) times the original size of the beam. The asymmetrically cut MC also reduces the beam divergence, which now becomes *w*_*i*_ *b*, where *w*_*i*_ is the divergence of incident beam. Thus the factor *b* is related to both the beam divergence and expansion.

The expanded X-ray beam is incident on a sample where it is subject to absorption and refraction by the sample. The X-ray beam exiting the sample strikes the LA and is split into a forward-diffracted beam and a diffracted beam. Both forward-diffracted and diffracted beams undergo intensity modulation according to their corresponding rocking curves. In this study, we employed only the forward-diffracted beam, detected by a CCD camera. The schematic of the XDFI system used in the research is shown Fig 2.

**Fig 3:**
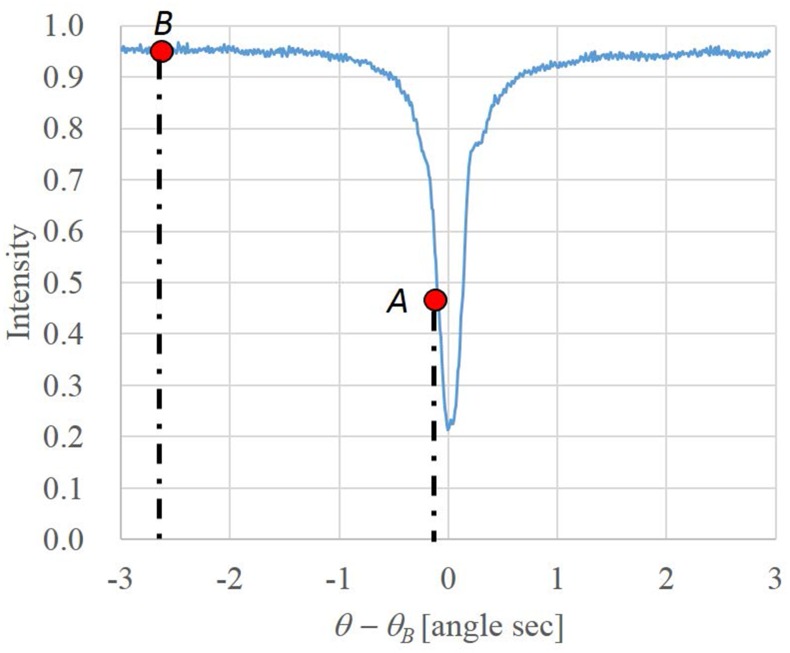
The rocking curve for the forward-diffraction.

Fig 3 shows an example of rocking curve for the forward-diffracted beam under the condition of silicon 440 diffraction and 35keV energy of X-rays. The horizontal axis of this graph is the incident angle measured from the Bragg angle of the analyser while the vertical axis shows the transmissivity of the LA Laue crystal. The full width at half maximum of the rocking curve in our experiments was approximately 2×10^−6^ radians. Since the variation of refraction angle in soft tissue with hard X-ray region is approximately 10^−7^ radians, it be converted to X-ray intensity by slope of rocking curve as shown in the point *A* in Fig 3.

In flat area of rocking curve, as denoted by point *B*, the intensity modulation does not occur. Therefore, at point *B* we obtain a projection image that contains only the absorption information. On the other hand, observed refraction modulated image is a mixture of the refraction and absorption components of the sample. The pure refraction component is acquired by dividing the refraction-modulated image by the absorption image.

### Artefact Reduction Reconstruction Algorithm

In order to reconstruct an artefact-free phase-contrast CT, we use an iterative reconstruction algorithm that goes back and forth between the projection data set and its phase-contrast tomographic image (i.e., between the sinogram and its corresponding tomogram) while imposing a priori information on both. A step-by-step description of this algorithm is given in [16]. For completeness, we describe the main intuition behind this algorithm.

#### Step 1

**Fig 4:**
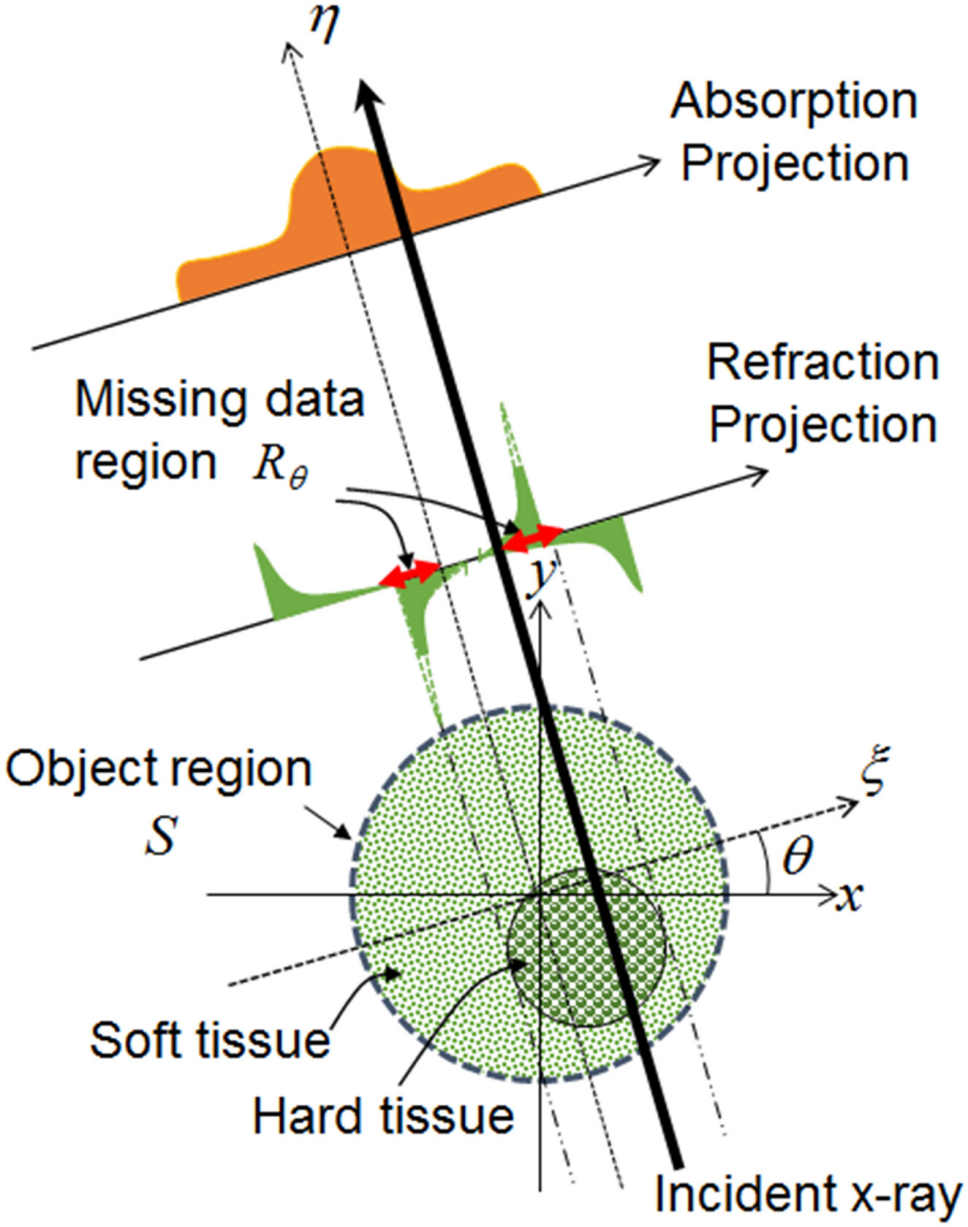
The main intuition behind the artefact reduction algorithm. Since the missing data is present only at the edges of dense objects, one can use the absorption image to localize the regions of non-linear effects responsible for the artefacts.

The first step in this algorithm is to separate out the differential refraction component from the measured projections that contain both absorption and refraction components; this gives us a pure refraction sinogram *p* ^(0)^(*ξ*,*θ*). Separately, we reconstruct an absorption-contrast tomographic image from the measured absorption projections. Using a threshold to separate the soft and dense objects, we isolate regions containing dense structures such as bones and calcifications that are responsible for a large beam-deflection. Since these regions have incorrect phase information, we regard them as areas of missing data in each differential refraction projection and set their pixel values as 0 to estimate them in the following steps. The end result is the specification of the missing-data region *R*_*θ*_ in the differential refraction projection *p* ^(0)^(*ξ*,*θ*) for each *θ*. We compute the object region *S* from the phase-contrast tomogram using the conventional algorithm [17] (Fig 4).

#### Step 2

Feed an initial tomogram *f* ^(1)^(*x*,*y*), reconstructed with conventional method [17] and then smoothed to maximally reduce artifacts.

#### Step 3

Transform a tomogram updated *n* times, *f* ^(n)^(*x*,*y*), to the sinogram, *p* ^(n)^(*ξ*,*θ*), for each *θ* by performing line-integrals along each x-ray flux according to the imaging geometry (i.e., the Radon transform) and then differentiate them with respect to *ξ*, where *ξ* is an axis perpendicular to a direction of the x-ray flux (Fig 4), that is,

p(n)(ξ,θ)=∂∂ξ(ℜθf(n))(ξ,θ), (1)

where ℜ_*θ*_ is a projection operator for *θ*.

#### Step 4

First, replace the data in the region except the missing-data region, ${\bar{R}}_{\theta }$, with the corresponding data in *p* ^(0)^(*ξ*,*θ*). Next, impose to the differential refraction projections the condition that the total sum of the differential projection should be zero for each *θ* in terms of the following mathematical fact: Considering a general smooth function *φ*(*u*) with $\underset{|u|\to \infty }{\text{lim}}\varphi (u)=0$,

$$\varphi (v)=\int_{0}^{\varphi }d\varphi =\int_{-\infty }^{v}\frac{d\varphi }{du}du.$$ (2)

Therefore, $\int_{-\infty }^{\infty }\frac{d\varphi }{du}du=0$, because *φ*(∞) = 0. From the mathematical theorem, since the Radon transform of *f*(*x*,*y*) with a support, (ℜ_*θ*_ *f*)(*ξ*,*θ*), always satisfies $\underset{|\xi |\to \infty }{\text{lim}}({ℜ}_{\theta }f)(\xi ,\theta )=0$,

$$\int_{-\infty }^{\infty }p(\xi ,\theta )d\xi =\int_{-\infty }^{\infty }\frac{\partial }{\partial \xi }({ℜ}_{\theta }f)(\xi ,\theta )d\xi =0$$ (3)

for each *θ*. Thus, the total sum of the differential projection should be zero for each *θ*. From the above discussion, for each *θ*,

$${\tilde{p}}^{\left(n\right)}(\xi ,\theta )=\{\begin{array}{ccc} p^{\left(0\right)}(\xi ,\theta ) & \text{for} & \xi \in {\bar{R}}_{\theta }\\ k\;p^{\left(n\right)}(\xi ,\theta ) & \text{for} & \xi \in R_{\theta } \end{array},$$ (4)

where *k* satisfies

$$\int_{{\bar{R}}_{\theta }}^{}p^{\left(0\right)}(\xi ,\theta )d\xi +k\int_{R_{\theta }}^{}p^{\left(n\right)}(\xi ,\theta )d\xi =0.$$ (5)

#### Step 5

Transform the updated refraction sinogram,${\tilde{p}}^{\left(n\right)}(\xi ,\;\theta )$, to the tomogram, ${\tilde{f}}^{\left(n\right)}(x,\;y)$, by convolving it with the signum function for each *θ* and then applying Algebraic Reconstruction Technique (ART); that is,

$${\tilde{f}}^{\left(n\right)}(x,\;y)=ℑ\;\;\left(\text{sgn}⊗{\tilde{p}}^{\left(n\right)}\right)(x,\;y),$$ (6)

where ℑ is a reconstruction operator.

#### Step 6

Replace the pixel value in the object region *S* representing the soft tissues, and then remove noise and streak artefact using the total variation; in the region $\bar{S}$ where the missing data from dense edges is in the beam path, a pixel value of zero is substituted, i.e.,

f˜' (n)(x, y)={f˜(n)(x, y)for (x, y)∈S0for (x, y)∈S¯. (7)

In the original algorithm described in [16], the result of Eq (7) was directly fed into Step 3 as it is. However, Step 3 requires numerical differentiation of the projections obtained by Radon transforming the result of Eq (7). Since noise is amplified by numerical differentiation, there is a need to effectively remove noise in this step. In order to remove the accumulation noise, we add the following de-noising process based on the total variation (TV) regularization [18], and set *n* = *n* + 1:

f(n+1)(x, y)=arg min f(x,y)≥0‖f−f˜' (n)‖2+λ TV(f), (8)

where *λ* is a Lagrange multiplier, and

$$TV(f)=∭\;\sqrt{\;{\left(\frac{\partial f}{\partial x}\right)}^{2}+\;{\left(\frac{\partial f}{\partial y}\right)}^{2}}\;dxdy.$$ (9)

#### Step 7

Iterate Steps 3 through 6 until $\left\|f^{\left(n+1\right)}(x,\;y)-f^{\left(n\right)}(x,\;y)\right\|\;/\;\left\|f^{\left(n\right)}(x,\;y)\right\|$ converges to a predefined value (e.g., 1.0×10^−3^).

**Fig 5:**
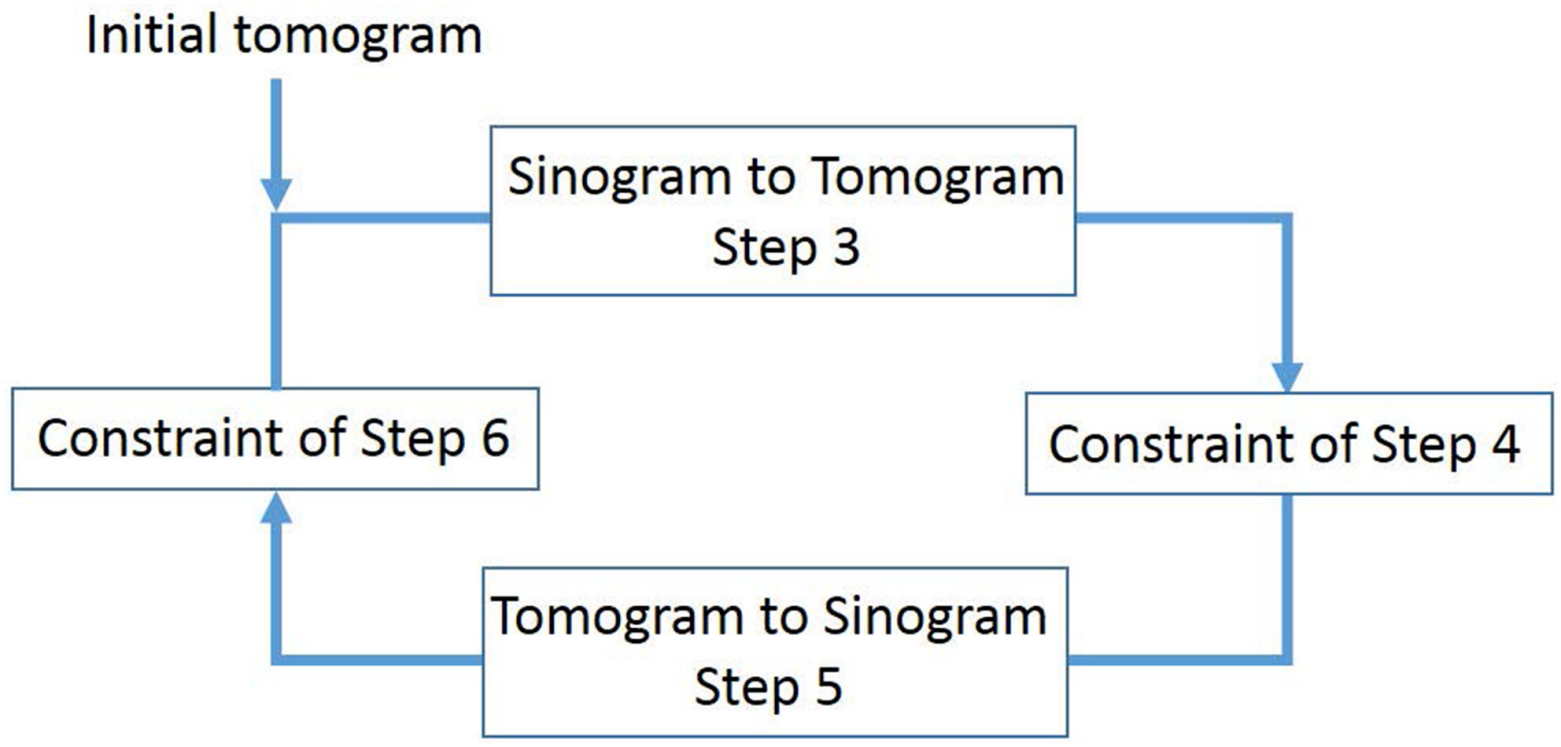
A flowchart of the main computational steps in the artefact reduction algorithm.

A flowchart for the main computational steps of the above algorithm is given in Fig 5. This algorithm is similar to Papoulis’s algorithm to extrapolate a signal from the band-limited condition [19] and Fienup’s algorithm to retrieve phase information from an intensity signal [20]. These are iterative algorithms that go back and forth between the signal and the transformed domains, via Fourier and inverse-Fourier transforms, while imposing *a-priori* information on individual spaces. By virtue of the Fourier slice theorem [21], the sinogram has a one-to-one correspondence with the two-dimensional (2D) Fourier space; therefore, the proposed algorithm may be regarded as a variant of Papoulis’s or Fienup’s algorithm.

### Biological Specimen Preparation

In this study, human specimens containing atherosclerotic disease and an animal model of rheumatoid arthritis were scanned. The request to image ex-vivo, formalin-fixed tissue specimens was reviewed by the Institutional Review Board (IRB) at the Massachusetts General Hospital. The IRB waived the needed for a formal IRB application because the tissue specimens are non-traceable and anonymous and were obtained from discarded human tissue during routine autopsy procedures.

The atherosclerotic disease specimens consisted of approximately 1 cm segments of human iliac and coronary arteries. The specimens were fixed in neutral 20% formalin for scanning.

Adjuvant arthritis, as a model of human rheumatoid arthritis, was induced in 8-week-old female Lewis rats (Charles River Laboratories Japan Inc., Kanagawa, Japan). This was accomplished via a single intradermal injection, into the footpad of the right hind paw, of 0.1 ml Freund’s complete adjuvant (Wako Pure Chemical Industries, Osaka, Japan) containing 0.5 mg heat-killed *M*. *tuberculosis* H37Ra emulsified in liquid paraffin (Wako Pure Chemical Industries). As the arthritis developed in this joint, it was amputated 21 days after the induction; the un-injected normal left leg was also resected above the ankle joint on day 22. The joints were fixed in neutral 20% formalin. The specimens were installed in a plastic tube filled with agarose gel for scanning. The overall size of the specimen was approximately 23×23×35 mm^3^.

All procedures using the rat were in accordance with the "Guidelines for Animal Experimentation of the Japanese Association for Laboratory Animal Science," and were approved by the Animal Use and Care Committee at Mercian Cleantec Corporation.

### Data Acquisition on the XDFI System

#### Iliac and Coronary Artery Specimens

A monochromatic X-ray beam with an energy of 31 keV and beam area of approximately 70×40 mm was used for imaging. The MC used was an asymmetric Bragg-case Si (440) crystal with *θ*_*B*_ = 12.0 degree at 31 keV, *α* = 11.1 degree and thus *b* = 0.04. The LA used was a symmetric Laue-case Si (440) crystal with *θ*_*B*_ = 12.0 degree at 31 keV and 150 μm thickness. Once again, a water-cooled CCD camera (Photonic Science, Ltd.) with a dynamic range of 12 bits was used. Its pixel size was 7.4 μm and it had a field of view 36.1 mm (h)×24.0 mm (v).

For the CT reconstruction of the iliac artery, 360 refraction images, and an equal number of absorption images, were obtained while rotating the sample around a vertical axis. The angular interval was 0.5 degree, and the angular span was 180 degree. It took approximately 5s to acquire each projection image for the iliac artery specimen and the total data acquisition time for one tomogram was approximately 1 hour.

For the CT reconstruction of the coronary artery, a finer angular interval of 0.3 degrees between projections was used resulting in 600 projections each for refraction and absorption images over a 180-degree angular span. It takes approximately 6 s to acquire each projection image and the entire data set consisting of 600 projection images took approximately 2 hours to acquire.

#### Rat Foot

The data were acquired using monochromatic X-ray beam with an energy of 35 keV and beam area of 70×40 mm. For the MC, the XDFI setup used a silicon crystal, asymmetrically cut in the Bragg-case, with Si (440) crystal plane with Bragg angle *θ*_*B*_ = 10.6 degree at 35 keV, asymmetric angle *α* = 10.2 degree and thus, asymmetric factor *b* = 0.02. The analyser LA employed a symmetric Laue-case Si (440) crystal plane with *θ*_*B*_ = 10.6 degree at 35 keV and 1.5 mm thickness. A water-cooled CCD camera (Photonic Science, Ltd.) was used for recording the projection images; it had a 12.5 μm pixel size, 12-bit dynamic range, and a reasonably large field of view of 49 mm (h)×33.0 mm (v). For the CT reconstruction, 900 projections at refraction and absorption locations each were obtained while rotating the sample around a vertical axis. The angular interval was 0.2 degree, and the angular span was 180 degree. It took approximately 2 seconds to acquire each projection image and total data acquisition time for one tomogram was approximately 1 hour.

## Results

### Effectiveness of de-noising procedure

**Fig 6:**
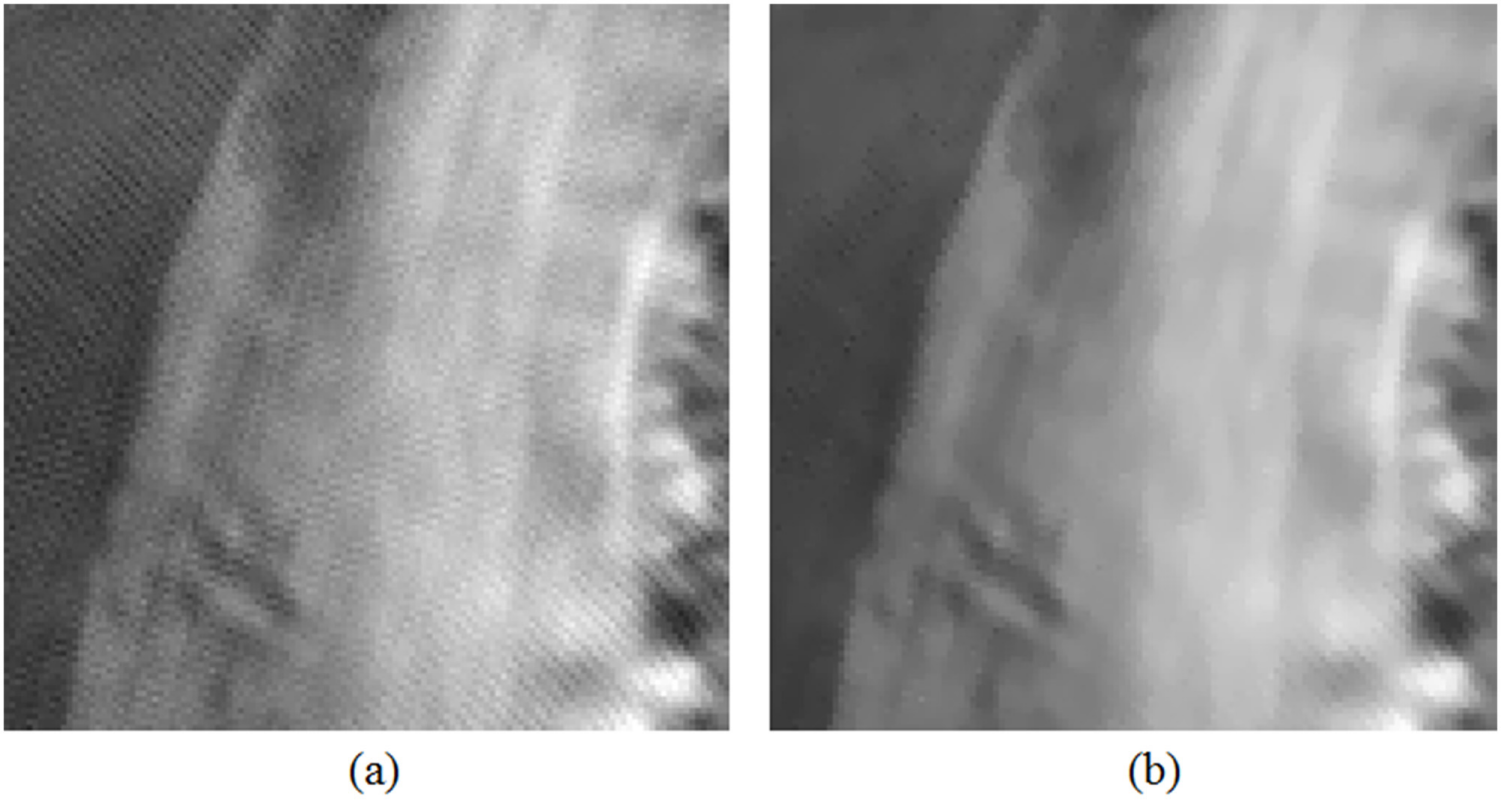
Comparison between the algorithms proposed in reference [16] and that described in the current paper. Panel (a): Phase-contrast images reconstructed using the algorithm without any de-noising step, as proposed in reference [16]; Panel (b): reconstructed image using the de-noising procedure proposed in this research. Both panels are zoomed-in detail of the region indicated by a red dashed square in the top image in Fig 7(c).

**Fig 7:**
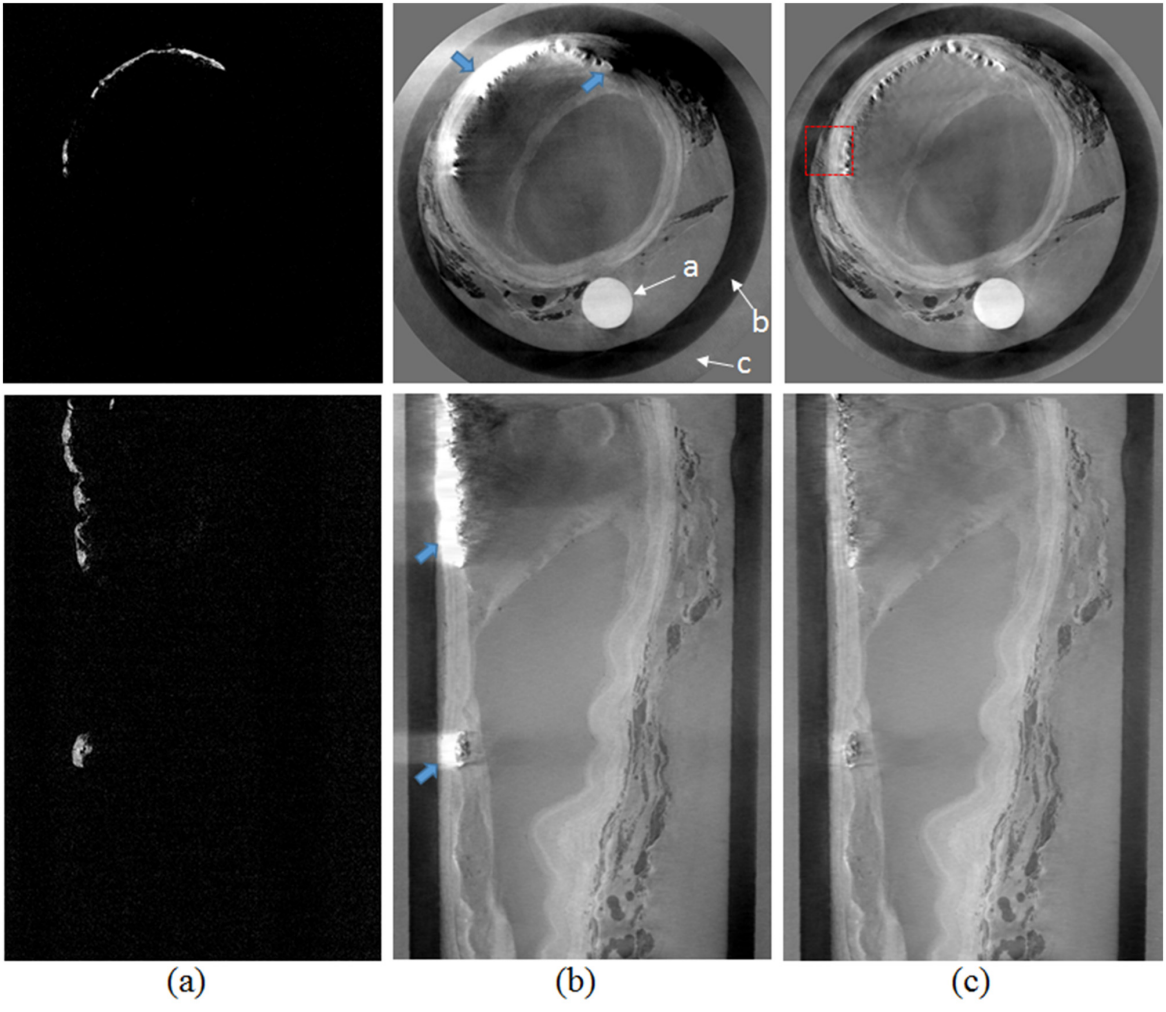
Iliac artery specimen in cross-sectional (top) and longitudinal (bottom) cut planes. Absorption-contrast images (a), phase-contrast images with calcification artefact (b), and phase-contrast image using the proposed artefact removal algorithm (c) are shown.

We investigated the effectiveness of the de-noising procedure by comparing images reconstructed with and without the de-noising step. Fig 6(a) and 6(b) show the zoomed-in sub-region of a human iliac artery specimen, indicated by the red dashed square in Fig 7(c), reconstructed with and without the de-noising procedure, respectively. Although the minute and high-frequency artefacts are observed in Fig 6(a), they are effectively reduced in Fig 6(b).

### Atherosclerotic Plaque Specimens

Fig 7 shows cross-sectional and longitudinal slices from reconstructed CT images with the absorption-contrast (a), phase-contrast without artefact removal (b), and phase-contrast by the proposed method with artefact removal(c). In this figure, ‘a’ denotes an embedded acrylic rod used for holding the sample, ‘b’ is the plastic container, and ‘c’ is the water bath for the specimen. In the absorption-contrast image (Fig 7(a)), only the dense calcifications that are primarily responsible for the beam deflection artefacts are visible. In the phase-contrast image with calcification artefact (Fig 7(b))—even though it shows the atherosclerotic plaque components in considerably better detail than the absorption-contrast image---the contrast is limited due to beam deflection artefacts indicated by blue arrows. These artefacts make the soft tissue in the vicinity of the calcifications invisible, a condition that is dramatically improved by the proposed algorithm (Fig 7(c) and S1 Movie).

**Fig 8:**
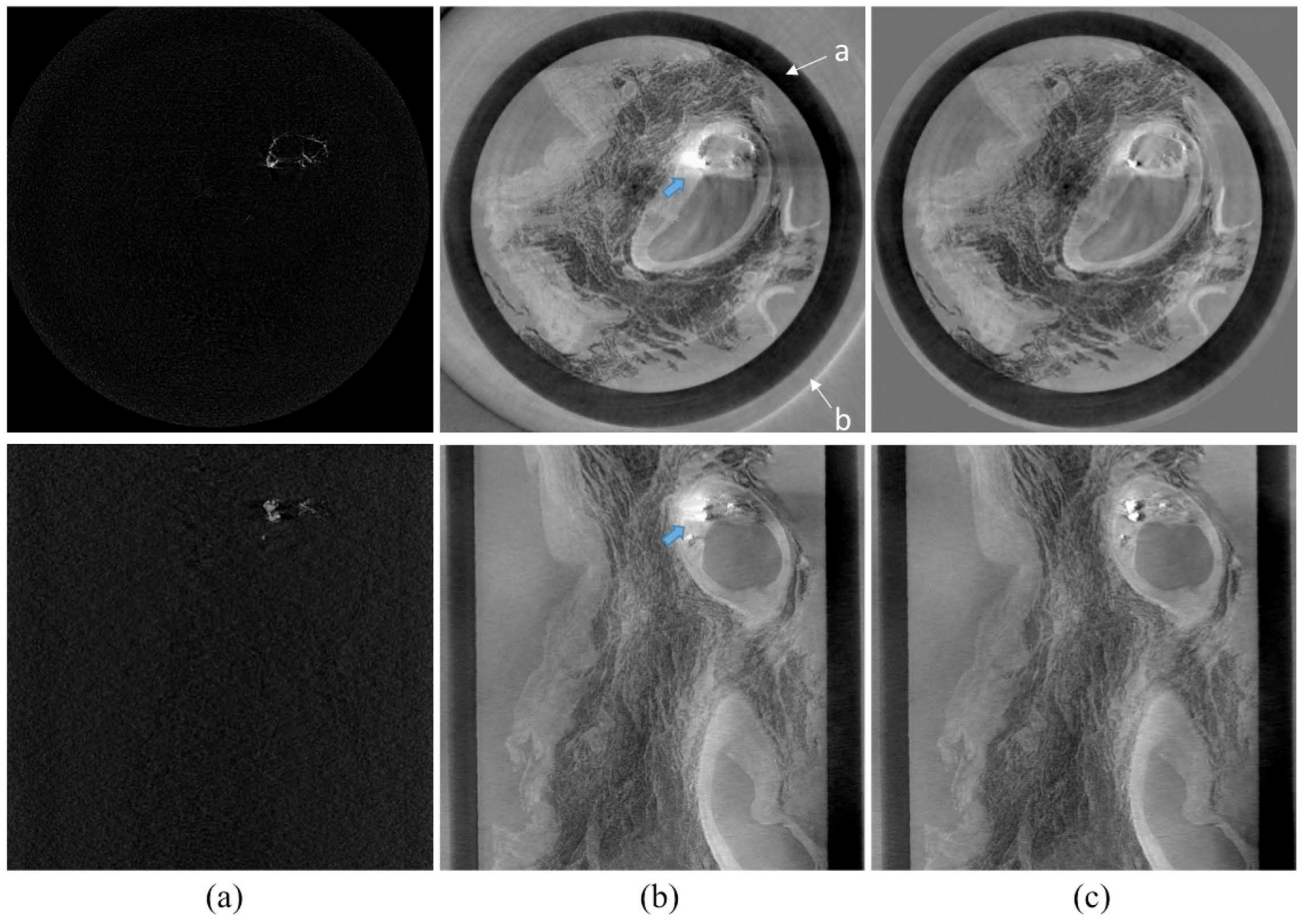
Coronary artery specimen in cross-sectional (top) and longitudinal (bottom) cut planes. Absorption-contrast images (a), phase-contrast images with calcification artefact (b), and phase-contrast image using the proposed algorithm (c) are shown.

Fig 8 shows similarly reconstructed CT images of a human coronary artery with calcified atherosclerotic plaque. The organization of this figure is similar to that in Fig 7, ‘a’ marks the plastic container and ‘b’ denotes the water bath for the specimen. Once again, the absorption-contrast images (Fig 8(a)) only shows calcifications while the phase-contrast images (Fig 8(b) and 8(c)) illustrate the components of the plaque. As seen in the previous images, the proposed algorithm considerably reduces the beam deflection artefacts (Fig 8(c) and S2 Movie).

### Arthritis

**Fig 9:**
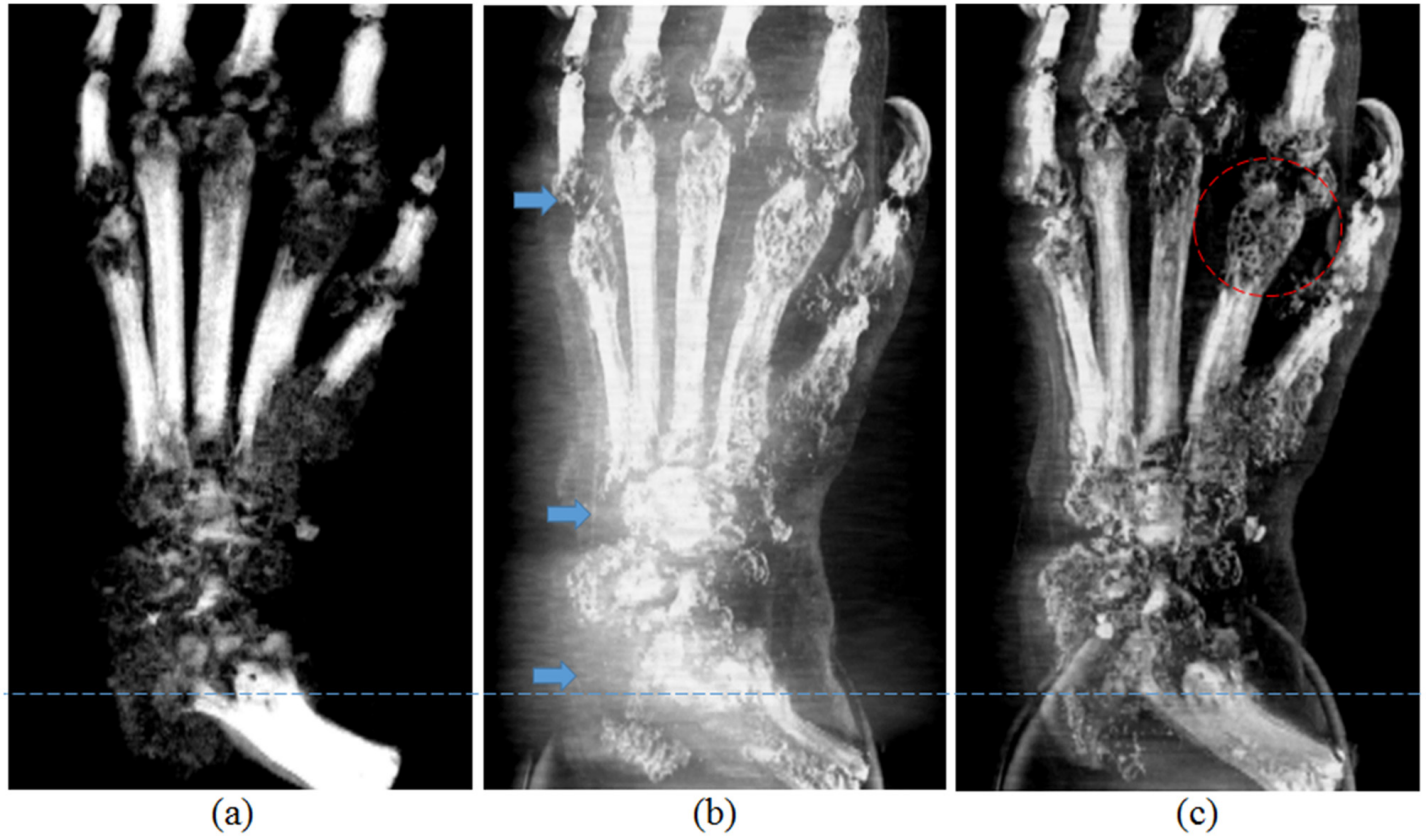
MIP images from a rat model of human rheumatoid arthritis. Absorption (a), phase contrast with bone artefact (b), and phase contrast using the proposed algorithm (c).

Fig 9 shows maximum intensity projection (MIP) images of the arthritic rat foot using absorption-contrast (a), phase-contrast with bone artefact (b), and phase-contrast with bone artefact removed using the proposed algorithm (c), respectively. As expected, the absorption-contrast image (Fig 9(a)) only shows the bony details and has very little contrast in the bone destroyed by arthritis. The arthritic areas around the joints are simply shown as radiolucent areas due to their low absorption with little or no internal detail. On the other hand, the phase-contrast images (Fig 9(b) and 9(c)) show the detailed morphology of the affected joints and the surrounding soft tissues up to the skin surface (dashed circle).

**Fig 10:**
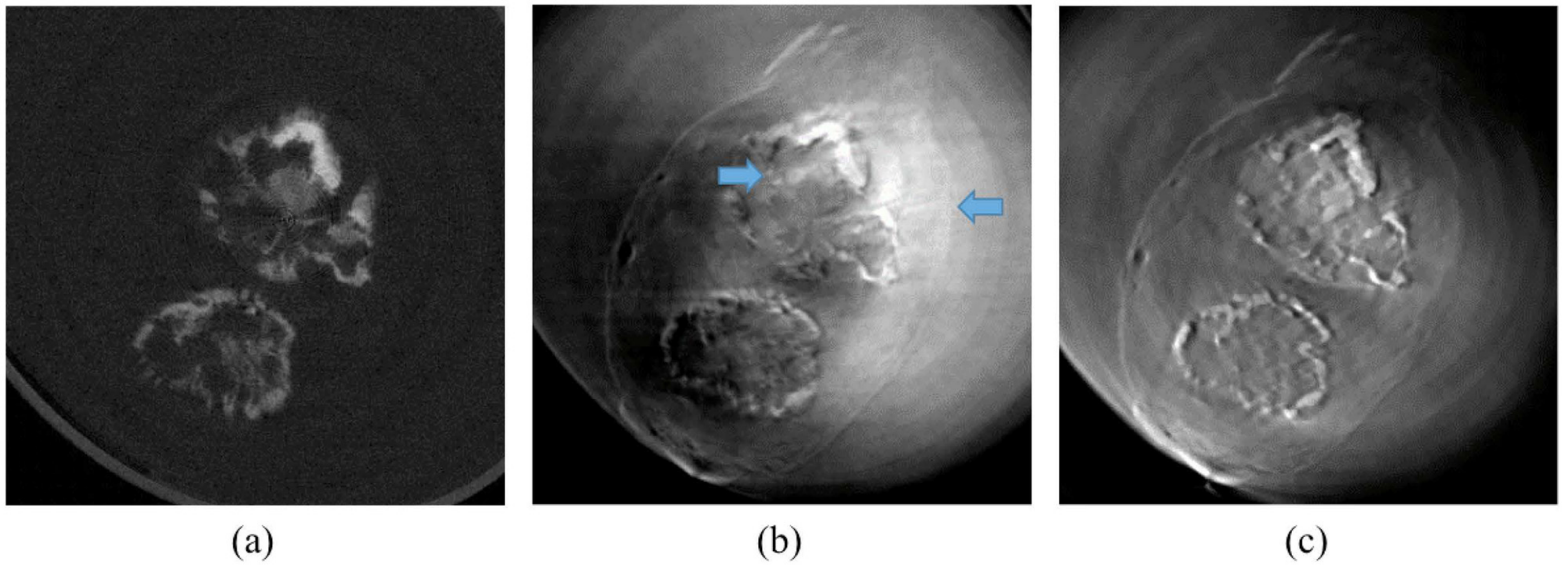
A cross-sectional slice at the level of the dashed line in Fig 8. Note the considerable reduction in beam deflection artefacts in (c) as compared to (b).

This figure also demonstrates the areas of signal loss due to phase large change in beam direction by dense structures. As can be seen in the regions marked by arrows in Fig 9(b), there is near complete signal drop out due to artefacts. The proposed algorithm dramatically improves the detail within these regions (Fig 9(c) and S3 Movie). A cross sectional slice located at the dashed line in Fig 9 is shown in Fig 10, where the parts (a), (b), and (c) correspond to those in Fig 10, respectively. As can be seen, the areas of signal dropout in the vicinity of dense objects are restored by the proposed algorithm.

## Discussion and Conclusion

It is widely acknowledged that phase contrast imaging improves the low-contrast resolution of images. This feature is especially useful when visualizing soft-tissue structures that are composed of materials with very similar atomic number. This paper confirms this result and shows that X-ray phase contrast imaging can be used to better advantage when studying rheumatoid arthritis or atherosclerosis.

A limitation of existing methods of phase contrast imaging is that it is prone to artefacts in the presence of dense structures. The key observation in this paper is that these structures can be localized and segmented out using the absorption image where they have high CT numbers. Because the phase and absorption projections are registered with each other, the pixels that are affected by them are well localized both on both types of images. By setting these artefact generating voxels as unknown, and using an iterative reconstruction algorithm, one can constrain and estimate their value from the projections provided. This algorithm was implemented and tested on a phase-contrast imaging system built using coherent radiation from a synchrotron source.

Human atherosclerotic plaque specimens and a model of rheumatoid arthritis implemented in a rat foot were used to test the efficacy of this artefact suppression algorithm. Atherosclerotic plaque is composed of artefact producing dense calcifications as well as fine soft-tissue components such as the fibrous cap and atheroma that are important for disease characterization. Similarly, the rat foot specimen is excellent for demonstrating the effectiveness of the proposed method because it consists of a mixture of low-density bone fragments that have been destroyed by adjuvant-induced arthritis and high-density healthy bones. Clear demarcation of these structures is important in order to assess the extent of disease. Our results demonstrate that the proposed algorithm is quite effective in suppressing artefacts arising from dense structures and is able to show soft-tissue detail in close proximity of such structures. As result, our methodology may be better suited for assessing the extent of disease or response to therapy than the traditional absorption- or phase-contrast images.

In closing, some limitations of the current study should be pointed out. First, it was conducted using a small number of specimens and disease states. As such, the current study should be viewed as a demonstration rather than a proof of the efficacy of the proposed algorithm. The algorithm was demonstrated for the X-ray dark-field imaging setup. Its efficacy for other phase-contrast imaging methods such as those based on Talbot interferometry and gratings [3, 7] remains unknown.

Since the setup used for this research requires a synchrotron source, another limitation of the proposed method is that it is not well suited for directly imaging patients because of radiation dose considerations. Nonetheless, the current setup can be used to advance our knowledge of disease by imaging biopsy specimens and other such human tissue. It should be noted that the proposed algorithm is a general technique that is applicable to all phase contrast imaging setups: the only requirement is that they yield a registered pair of phase and attenuation images. Therefore, it may be applicable to other phase contrast imaging techniques. For example, recent advances phase-contrast imaging optics have enabled grating interferometry to acquire the phase-contrast CT using a standard x-ray source. Since these setups also provide registered sets of phase and attenuation projections, the proposed algorithm should be applicable to these setups.

## Supporting Information

S1 Movie MIP movie of iliac artery specimen. (MP4)

S2 Movie MIP movie of coronary artery specimen. (MP4)

S3 Movie MIP movie of a rat model of human rheumatoid arthritis (MP4)
